# Benchmarking Deep Learning-Based Image Retrieval of Oral Tumor Histology

**DOI:** 10.7759/cureus.62264

**Published:** 2024-06-12

**Authors:** Ranny R Herdiantoputri, Daisuke Komura, Mieko Ochi, Yuki Fukawa, Kou Kayamori, Maiko Tsuchiya, Yoshinao Kikuchi, Tetsuo Ushiku, Tohru Ikeda, Shumpei Ishikawa

**Affiliations:** 1 Department of Oral Pathology, Tokyo Medical and Dental University, Tokyo, JPN; 2 Department of Preventive Medicine, The University of Tokyo, Tokyo, JPN; 3 Department of Pathology, Teikyo University School of Medicine, Tokyo, JPN; 4 Department of Pathology, The University of Tokyo, Tokyo, JPN

**Keywords:** convolutional neural networks, histopathology diagnosis, medical image, oral tumor, oral pathology, machine learning

## Abstract

Introduction: Oral tumors necessitate a dependable computer-assisted pathological diagnosis system considering their rarity and diversity. A content-based image retrieval (CBIR) system using deep neural networks has been successfully devised for digital pathology. No CBIR system for oral pathology has been investigated because of the lack of an extensive image database and feature extractors tailored to oral pathology.

Materials and methods: This study uses a large CBIR database constructed from 30 categories of oral tumors to compare deep learning methods as feature extractors.

Results: The highest average area under the receiver operating characteristic curve (AUC) was achieved by models trained on database images using self-supervised learning (SSL) methods (0.900 with SimCLR and 0.897 with TiCo). The generalizability of the models was validated using query images from the same cases taken with smartphones. When smartphone images were tested as queries, both models yielded the highest mean AUC (0.871 with SimCLR and 0.857 with TiCo). We ensured the retrieved image result would be easily observed by evaluating the top 10 mean accuracies and checking for an exact diagnostic category and its differential diagnostic categories.

Conclusion: Training deep learning models with SSL methods using image data specific to the target site is beneficial for CBIR tasks in oral tumor histology to obtain histologically meaningful results and high performance. This result provides insight into the effective development of a CBIR system to help improve the accuracy and speed of histopathology diagnosis and advance oral tumor research in the future.

## Introduction

Oral tumors are generally composed of diverse and rare tumor types, except for major categories like squamous cell carcinoma. Distinguishing oral tumor types is difficult except for well-experienced oral pathologists. The rarity of oral tumors and the diverse tissue types in the oral region make obtaining reference images for diagnosis and research a challenge, potentially leading to delayed diagnosis and a significant burden on pathologists [1]. Consequently, a diagnostic system is needed to improve the speed and accuracy of histopathological diagnosis of these tumors [2]. Artificial intelligence (AI) is a promising solution for efficient histopathological diagnosis of oral tumors.

AI development for oral tumor diagnosis is limited and focused only on a few tumor types. Classification methods have been developed to predict the diagnosis, such as differentiating between ameloblastoma or odontogenic keratocysts, to which a histopathological image may belong [3,4]. These approaches are helpful in common cases. However, a computer-aided diagnostic system, specifically for histological images, which covers a broader spectrum of tumor types would be more practical and help narrow the differential diagnoses. Therefore, content-based image retrieval (CBIR) is suitable. CBIR is a method of obtaining images that are relevant to a query image from a large collection of images based on their visual content. CBIR regards histopathological images as query images to find similar images from a database based on their similar morphology [2,5]. This system is useful as a diagnostic aid for finding case references, especially where diagnostic expertise is challenging to find, such as in low- to middle-income countries [1]. The involvement of human intervention is crucial in diagnosis. Conventionally, pathologists diagnose directly after hematoxylin and eosin (H&E)-stained slide analysis or optionally use different methods as diagnostic aids: referring atlases, consulting subspecialist experts, or conducting ancillary tests. An automatic image search can complement these options to expedite image reference search. With scarce pathological expertise, a tool that could provide urgently needed information for rapid diagnosis before conducting tests to raise a definitive one would be significant [6]. CBIR provides interpretability because it presents multiple candidate images, which is beneficial when distinguishing between categories based on histopathological images alone, which is challenging, such as when information on dental infections or radiographic findings is needed. With CBIR, the retrieved results are to be evaluated by pathologists, reducing the risk of misdiagnosis owing to inaccurate results, especially for categories with very similar histology.

The CBIR system consists of two aspects: image feature extraction and nearest-neighbor search. While nearest-neighbor search is implemented in the last step of image retrieval to locate the data points in high-dimensional space that is closest to the query point, feature extraction is implemented first and it is crucial because it must adequately capture complex histological features such as staining patterns, tissue structures, and cellular morphology to create histologically relevant image representation [2,7,8]. The extracted features must be robust to irrelevant color variations, such as different H&E stain brands, glass slide color degradation, and image-capturing devices ranging from whole-slide image (WSI) scanners to smartphone cameras [5,8,9]. At the early stage of CBIR development, traditional image features such as shape, color, texture, or a combination were used. Recent developments showed that deep learning models outperformed traditional features [6,7]. Several deep learning methods, such as supervised learning where models are pre-trained on general images or fine-tuned on histopathological images, have been used to train feature extractors [9-12], and self-supervised learning (SSL), which allows learning from unlabeled images [13-15]. However, no studies have reviewed which method is most suitable for CBIR in oral tumors.

This study aimed to investigate the performance of different deep learning models for oral tumor CBIRs by developing a large dataset of WSIs from 541 cases with 51 tumor types and evaluating the retrieval accuracy by comparing different representational learning techniques.

This article was previously posted to the medRxiv preprint server on May 31, 2024.

## Materials and methods

Dataset, database, and test queries

We collected diagnostic slides of the oral tumor categories described in chapters 7 and 8 of the WHO Classification of Head and Neck Tumours, 4th Edition [16] from Medical and Dental University (TMDU) Hospital (2001-2022) and scanned them to obtain the WSIs. Representative tumor regions were annotated, and image patches were randomly extracted. The dataset consists of 49,243 image patches from 51 categories, covering approximately 50% of the oral tumor categories (Table A1 in the Appendices).

**Figure 1:**
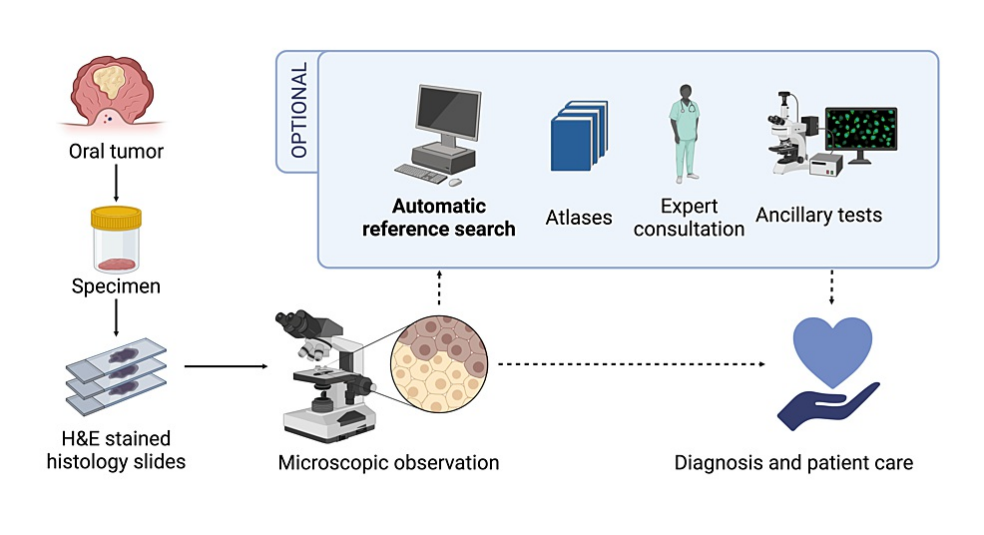
CBIR’s place in the pathological diagnosis workflow. CBIR optionally provides an interpretable automatic reference search that is fast and easily followed up with a more thorough study with atlases, discussions, or ancillary tests. CBIR could help point out similar features from the previously diagnosed image in the database that may lead to testable differential diagnoses more swiftly than directly consulting atlases or senior experts, which may cause the patient delayed treatment. CBIR: content-based image retrieval; H&E: hematoxylin and eosin. Image created with BioRender.

**Figure 2:**
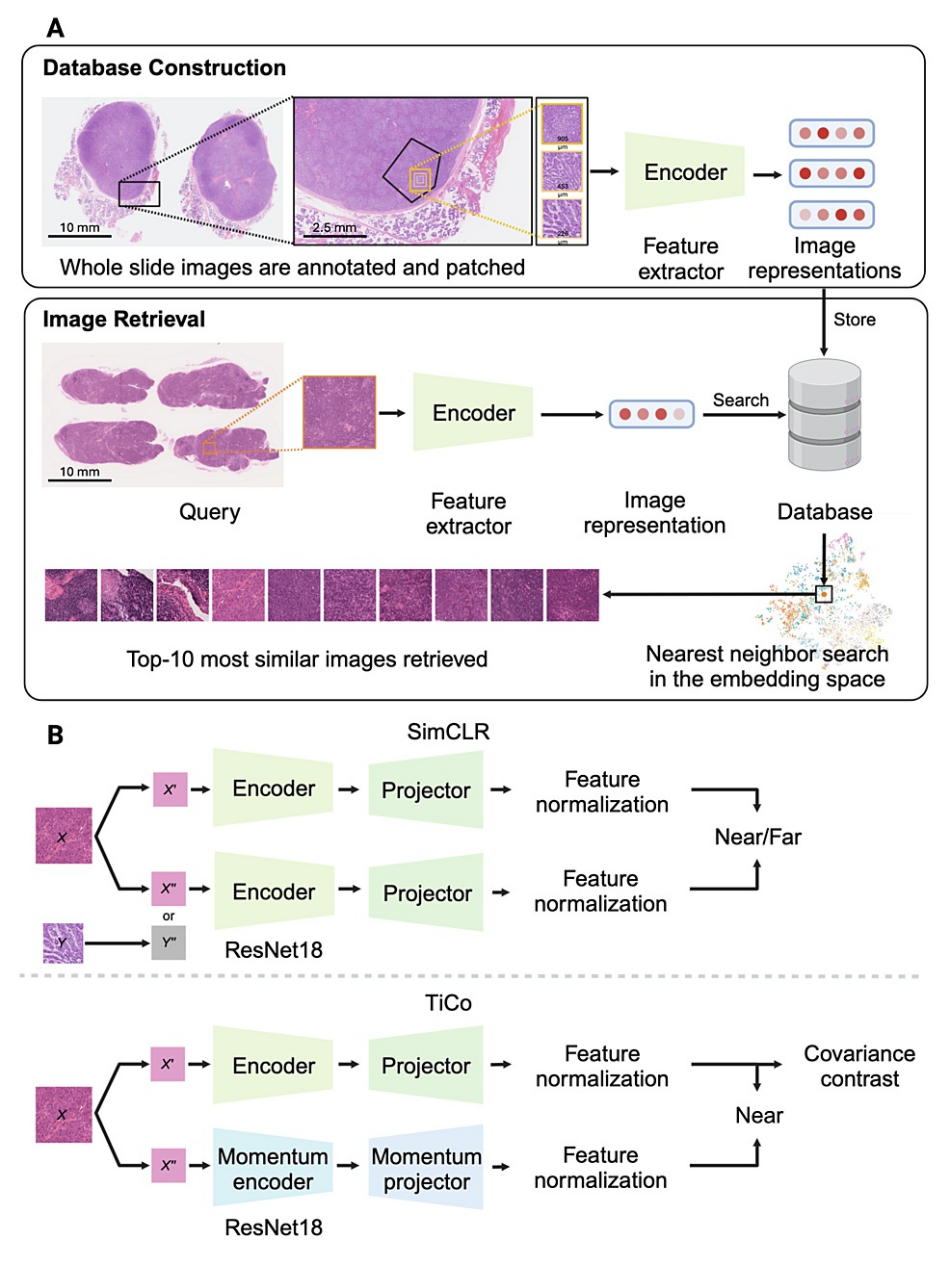
CBIR workflow and SSL models training process. (A) Schematic representation of CBIR using deep neural networks to retrieve similar oral tumor histopathological images. The similarity is determined by a nearest-neighbor search, which calculates the cosine similarity of the query image’s image representation to all database image representations in the embedding space. (B) The training process of the SSL models used ResNet18 as an encoder. The image representations were passed to a projector and subjected to feature normalization. In the SimCLR method, the training loss function yields a low value when the representation of the original image (X′) and its augmentation (X′′) are close together, while it yields a larger value when X′ and a different image augmentation (Y′′) are far apart. In the Transformation Invariance and Covariance Contrast (TiCo) method, the process still pulls X′ and X′′ close, and then the redundancy in the representation is removed using covariance contrast without using a different image (Y′′). CBIR: content-based image retrieval; SSL: self-supervised learning. Image created with BioRender.

A database from a subset of the dataset was compiled to develop an automated oral tumor image reference search (Figure 1). Image representations from each model’s encoder were stored in the database (Figure 2A). It contains 33,356 image patches from 30 oral tumor categories (Table A1 in the Appendices).

We prepared three query sets to test the performance. Query case set A was collected from TMDU Hospital. Histopathologic slides were scanned to create WSIs for in-domain queries. Three selected tumor areas that are typical of the tumor type from the same slides were photographed with smartphone cameras to create out-of-domain-phonecam queries. Query case set B (out-of-domain B) was compiled from the University of Tokyo Hospital and query case set C (out-of-domain C) was collected from Teikyo University Hospital. The number of images analyzed in each category is detailed in Tables A3-A5 in the Appendices.

The representation of each query image was calculated for each tested model. A nearest-neighbor search was performed based on cosine similarity with the database images. Examples of query images for each category in each set can be found in Figures A1-A3 in the Appendices. The details of the methods of dataset image collection, database construction, and test queries are available in Appendix Method 1. The database construction methods, including tumor area selection, patch extraction, feature extraction code, and image retrieval, were adapted from our previous study [17].

Evaluation metrics and statistical analysis

The area under the receiver operating characteristic curve (AUC) for all query images with top-k retrieved images (k ranges from 1 to the total number of cases in the database) being the cut-points were averaged into Mean-AUC. Based on the top 10 images most similar to the query, three additional metrics were evaluated. Mean-Acc denotes the mean of the top 10 diagnostic accuracies (Acc) for each query. %query denotes the percentage of results that contained at least one accurate diagnosis category. The histological similarity in the retrieved results beyond diagnostic accuracy was evaluated by noting the retrieved images that did not belong to the accurate diagnosis category or any of its differential diagnoses [16,18,19] (Table A2 in the Appendices). The values are expressed as histologic inaccuracy (HI) and were averaged to determine the mean HI. Image retrieval and all statistical analyses were conducted using Python 3.7.12 and R 4.2.2 (R Foundation for Statistical Computing, Vienna, Austria).

Seven deep learning models [13,20-25] were used to extract the features of each image patch, followed by CBIR performance evaluation. The model preparation and training method (Figure 2B) are available in Appendix Method 2.

## Results

The highest Mean-AUC for in-domain queries was achieved by ResNet18+SimCLR (0.900), followed by ResNet18+TiCo (0.897). They achieved this at the query category level for eight out of the 11 categories. The generalizability of these models was validated using out-of-domain-phonecam queries. The highest performance for out-of-domain-phonecam queries was also achieved by ResNet18+SimCLR (0.871), followed by ResNet18+TiCo (0.857). The highest performance on the query case category levels was achieved by both SSL models for seven out of the 11 categories. We tested the performance on queries from other institutions to further demonstrate the generalizability. A similar result was yielded by both SSL models, where ResNet18+SimCLR leads with the highest Mean-AUC (0.886 for out-of-domain-B and 0.913 for out-of-domain-D queries), followed by ResNet18+TiCo (0.881 for out-of-domain-B and 0.905 for out-of-domain-D queries). Phikon, pre-trained on histopathological images, and DINOv2, pre-trained on large-scale general images, performed comparably well with DINOv2 leading the overall Mean-AUC in out-of-domain-phonecam query, with Phikon leading in the other three query sets (Table 1).

**Table 1:** Mean-AUC (SD) of each test query category in in- and out-of-domain image queries. The performances of the SSL models are superior for most test query categories and the overall averages. The highest AUC for each category is marked in *italics*. SSL: self-supervised learning; AUC: area under the receiver operating characteristic curve.

Query	Category	Pre-trained VGG16	Pre-trained DINOv2	Fine-tuned ResNet18	Resnet18 + SimCLR	Resnet18 + TiCo	Ciga model	Phikon
In-domain (n = 120 images from 2 cases)	Nasopalatine duct cyst	0.758 (0.09)	0.815 (0.07)	0.684 (0.07)	0.857 (0.06)	0.855 (0.05)	0.811 (0.07)	0.812 (0.06)
	Glandular odontogenic cyst	0.842 (0.09)	0.890 (0.08)	0.770 (0.08)	0.899 (0.04)	0.899 (0.03)	0.832 (0.07)	0.817 (0.03)
	Odontogenic keratocyst	0.823 (0.07)	0.921 (0.04)	0.922 (0.04)	0.963 (0.03)	0.968 (0.03)	0.855 (0.07)	0.962 (0.02)
	Orthokeratinized odontogenic cyst	0.838 (0.08)	0.922 (0.10)	0.823 (0.10)	0.976 (0.03)	0.978 (0.03)	0.861 (0.07)	0.927 (0.04)
	Basal cell adenoma	0.937 (0.05)	0.905 (0.01)	0.973 (0.01)	0.961 (0.03)	0.961 (0.03)	0.924 (0.03)	0.954 (0.03)
	Adenoid cystic carcinoma	0.739 (0.12)	0.831 (0.05)	0.862 (0.05)	0.918 (0.05)	0.912 (0.07)	0.743 (0.11)	0.807 (0.12)
	Mucoepidermoid carcinoma	0.745 (0.11)	0.776 (0.07)	0.669 (0.07)	0.815 (0.09)	0.799 (0.08)	0.728 (0.13)	0.837 (0.05)
	Warthin’s tumor	0.966 (0.03)	0.939 (0.02)	0.968 (0.02)	0.997 (0.01)	0.996 (0.08)	0.945 (0.05)	0.990 (0.03)
	Odontogenic fibroma	0.730 (0.14)	0.805 (0.10)	0.642 (0.10)	0.769 (0.12)	0.763 (0.13)	0.695 (0.09)	0.755 (0.07)
	Ameloblastoma	0.730 (0.11)	0.730 (0.04)	0.664 (0.04)	0.809 (0.10)	0.808 (0.09)	0.701 (0.09)	0.744 (0.10)
	Hemangioma	0.886 (0.07)	0.842 (0.06)	0.881 (0.06)	0.935 (0.03)	0.929 (0.03)	0.842 (0.09)	0.886 (0.06)
	Average	0.818 (0.08)	0.852 (0.06)	0.805 (0.12)	0.900 (0.07)	0.897 (0.08)	0.812 (0.08)	0.863 (0.08)
Out-of-domain phonecam (n = 54 images from 2 cases)	Nasopalatine duct cyst	0.466 (0.08)	0.826 (0.05)	0.721 (0.07)	0.871 (0.04)	0.859 (0.02)	0.807 (0.06)	0.764 (0.05)
	Glandular odontogenic cyst	0.671 (0.10)	0.876 (0.04)	0.763 (0.06)	0.915 (0.02)	0.908 (0.02)	0.876 (0.03)	0.795 (0.04)
	Odontogenic keratocyst	0.622 (0.13)	0.891 (0.07)	0.614 (0.13)	0.961 (0.02)	0.968 (0.02)	0.892 (0.04)	0.907 (0.04)
	Orthokeratinized odontogenic cyst	0.647 (0.13)	0.891 (0.05)	0.751 (0.10)	0.920 (0.08)	0.917 (0.08)	0.875 (0.06)	0.939 (0.03)
	Basal cell adenoma	0.896 (0.07)	0.815 (0.07)	0.576 (0.14)	0.884 (0.08)	0.846 (0.09)	0.769 (0.08)	0.796 (0.08)
	Adenoid cystic carcinoma	0.779 (0.09)	0.793 (0.09)	0.634 (0.10)	0.850 (0.13)	0.799 (0.17)	0.651 (0.16)	0.652 (0.11)
	Mucoepidermoid carcinoma	0.536 (0.06)	0.700 (0.10)	0.562 (0.05)	0.828 (0.09)	0.793 (0.07)	0.724 (0.07)	0.707 (0.10)
	Warthin’s tumor	0.739 (0.09)	0.917 (0.06)	0.819 (0.08)	0.977 (0.04)	0.969 (0.06)	0.825 (0.14)	0.894 (0.08)
	Odontogenic fibroma	0.570 (0.12)	0.725 (0.11)	0.290 (0.05)	0.663 (0.13)	0.671 (0.12)	0.648 (0.10)	0.649 (0.10)
	Ameloblastoma	0.862 (0.04)	0.761 (0.10)	0.645 (0.04)	0.814 (0.12)	0.793 (0.11)	0.670 (0.10)	0.711 (0.06)
	Hemangioma	0.737 (0.17)	0.812 (0.05)	0.585 (0.10)	0.899 (0.06)	0.904 (0.05)	0.795 (0.11)	0.889 (0.05)
	Average	0.684 (0.13)	0.819 (0.07)	0.633 (0.14)	0.871 (0.08)	0.857 (0.9)	0.776 (0.09)	0.791 (0.10)
Out-of-domain-B	Myoepithelioma (n = 60 images from 1 case)	0.744 (0.08)	0.846 (0.05)	0.714 (0.05)	0.814 (0.05)	0.821 (0.04)	0.812 (0.05)	0.835 (0.05)
	Basal cell adenoma (n = 60 images from 1 case)	0.867 (0.09)	0.799 (0.07)	0.840 (0.10)	0.909 (0.04)	0.885 (0.08)	0.789 (0.10)	0.882 (0.06)
	Warthin’s tumor (n = 60 images from 1 case)	0.943 (0.04)	0.981 (0.02)	0.838 (0.07)	0.999 (0.01)	0.997 (0.02)	0.861 (0.05)	0.981 (0.01)
	Carcinoma ex pleomorphic adenoma (n = 60 images from 1 case)	0.766 (0.06)	0.808 (0.10)	0.684 (0.07)	0.913 (0.06)	0.903 (0.05)	0.774 (0.11)	0.865 (0.06)
	Mucoepidermoid carcinoma (n = 120 images from 2 cases)	0.797 (0.05)	0.822 (0.06)	0.619 (0.09)	0.814 (0.04)	0.802 (0.04)	0.745 (0.07)	0.770 (0.06)
	Adenoid cystic carcinoma (n = 120 images from 2 cases)	0.795 (0.08)	0.825 (0.05)	0.835 (0.05)	0.880 (0.08)	0.896 (0.09)	0.741 (0.07)	0.815 (0.06)
	Acinic cell carcinoma (n = 120 images from 2 cases)	0.779 (0.13)	0.803 (0.07)	0.759 (0.07)	0.802 (0.17)	0.789 (0.21)	0.660 (0.23)	0.816 (0.12)
	Salivary duct carcinoma (n = 60 images from 1 case)	0.904 (0.02)	0.889 (0.05)	0.798 (0.03)	0.961 (0.01)	0.953 (0.02)	0.864 (0.07)	0.952 (0.02)
	Average	0.825 (0.07)	0.847 (0.06)	0.761 (0.08)	0.886 (0.07)	0.881 (0.07)	0.781 (0.06)	0.864 (0.07)
Out-of-domain-C	Adenoid cystic carcinoma (n = 180 images from 3 cases)	0.846 (0.07)	0.807 (0.06)	0.812 (0.04)	0.887 (0.05)	0.871 (0.08)	0.774 (0.09)	0.842 (0.06)
	Basal cell adenoma (n = 180 images from 3 cases)	0.936 (0.04)	0.868 (0.08)	0.811 (0.06)	0.919 (0.05)	0.935 (0.04)	0.855 (0.06)	0.925 (0.03)
	Odontogenic myxoma/myxofibroma (n = 180 images from 3 cases)	0.899 (0.10)	0.935 (0.08)	0.784 (0.09)	0.970 (0.06)	0.962 (0.07)	0.955 (0.06)	0.914 (0.08)
	Fibrous dysplasia (n = 120 images from 2 cases)	0.811 (0.09)	0.850 (0.07)	0.759 (0.04)	0.907 (0.04)	0.848 (0.06)	0.813 (0.10)	0.824 (0.06)
	Osteoma (n = 180 images from 3 cases)	0.918 (0.09)	0.913 (0.09)	0.824 (0.04)	0.963 (0.05)	0.975 (0.03)	0.929 (0.07)	0.944 (0.08)
	Odontogenic keratocyst (n = 120 images from 2 cases)	0.806 (0.10)	0.893 (0.06)	0.540 (0.18)	0.871 (0.11)	0.875 (0.10)	0.837 (0.11)	0.878 (0.09)
	Orthokeratinized odontogenic cyst (n = 120 images from 2 cases)	0.758 (0.13)	0.907 (0.04)	0.634 (0.09)	0.949 (0.04)	0.932 (0.07)	0.836 (0.10)	0.895 (0.09)
	Adenomatoid odontogenic tumor (n = 120 images from 2 cases)	0.819 (0.07)	0.820 (0.08)	0.756 (0.04)	0.836 (0.05)	0.839 (0.05)	0.723 (0.13)	0.799 (0.09)
	Average	0.849 (0.06)	0.874 (0.09)	0.740 (0.09)	0.913 (0.04)	0.905 (0.05)	0.840 (0.07)	0.878 (0.05)

**Figure 3:**
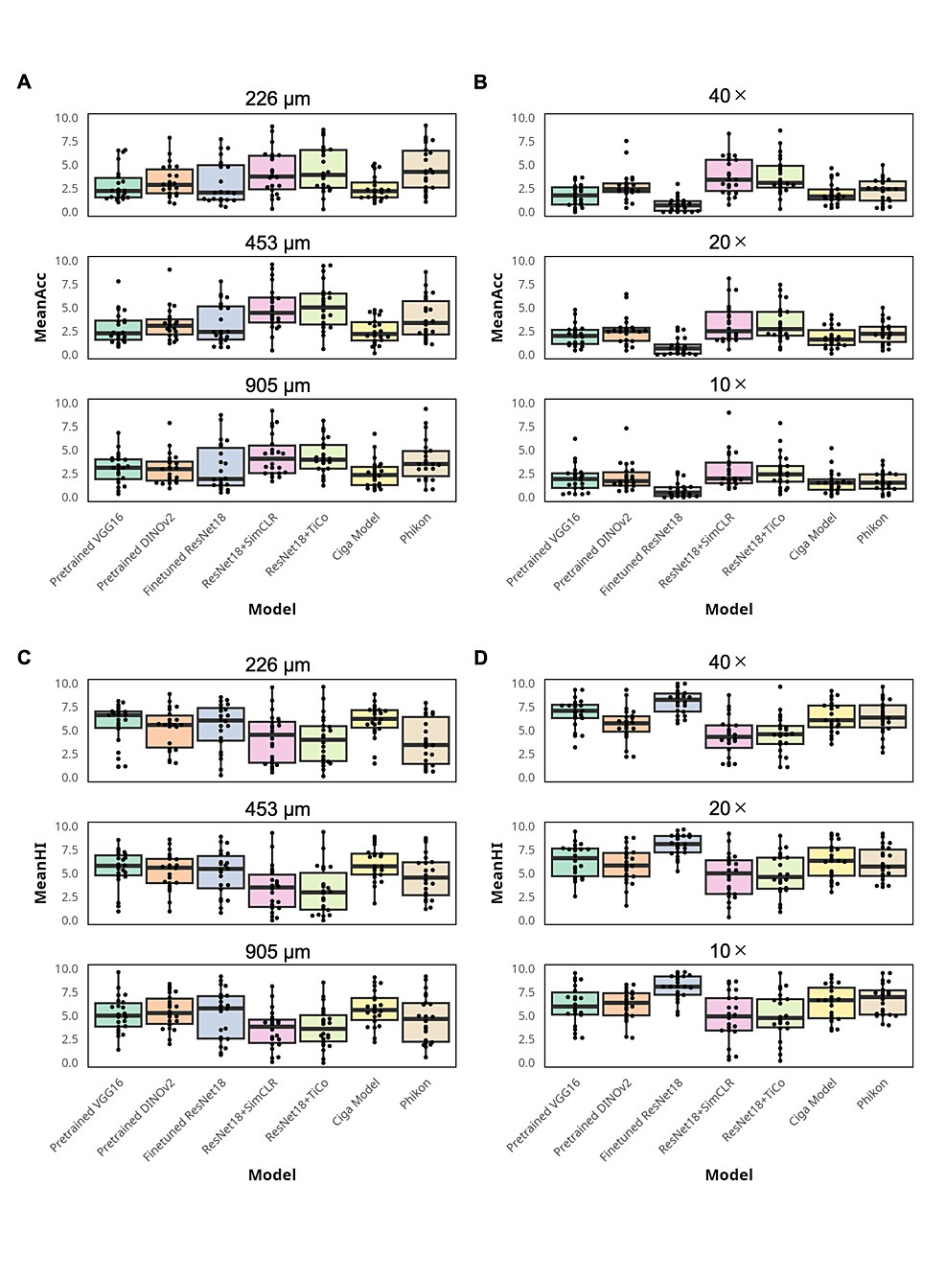
Mean-Acc and Mean-HI comparisons. (A) Mean-Acc comparison for the in-domain query of all models by magnification showing the highest performance of Phikon at the highest magnification and that of ResNet18+SimCLR and ResNet18+TiCo at the moderate and lowest magnification. (B) Mean-Acc comparison for out-of-domain-phonecam queries of all models by magnification shows the highest performance of ResNet18+SimCLR and ResNet18+TiCo at the highest and lowest magnification. Both model performances were comparable to that of pre-trained DINOv2 at moderate magnification. (C) Mean-HI comparison for in-domain queries by magnification shows that ResNet18+SimCLR and ResNet18+TiCo outperformed other models except for the highest magnification where Phikon leads with a wider interquartile range. (D) Mean-HI comparison for out-of-domain-phonecam query by magnification showing ResNet18+SimCLR and ResNet18+TiCo outperformed other models. (C-D) Please note that a lower Mean-HI value denotes a higher model performance. Acc: accuracies; HI: histologic inaccuracy.

Overall, the Mean-Acc of test query set-A was highest with the SSL models: ResNet18+TiCo outperformed other models for in-domain queries (4.64), followed by ResNet18+SimCLR (4.53) with no significant difference (Wilcoxon signed-rank test with Bonferroni adjustment). The reverse was observed for the out-domain-phonecam queries (ResNet18+SimCLR (3.33) and ResNet18+TiCo (3.31)) with no significant difference (Wilcoxon signed-rank test with Bonferroni adjustment). Phikon yielded the highest Mean-Acc for out-of-domain-B queries (3.79), followed by ResNet18+SimCLR (3.68). Pre-trained DINOv2 outperformed other models for out-of-domain-C queries (3.64), followed closely by ResNet18+TiCo (3.63) (Table A3 in the Appendices). The highest overall Mean-Acc was consistently achieved by the SSL models at different magnification levels, except for the high-magnification in-domain queries (Figures 3A, 3B). The highest %query was obtained with SSL models for most query categories (Table A4 in the Appendices).

**Figure 4:**
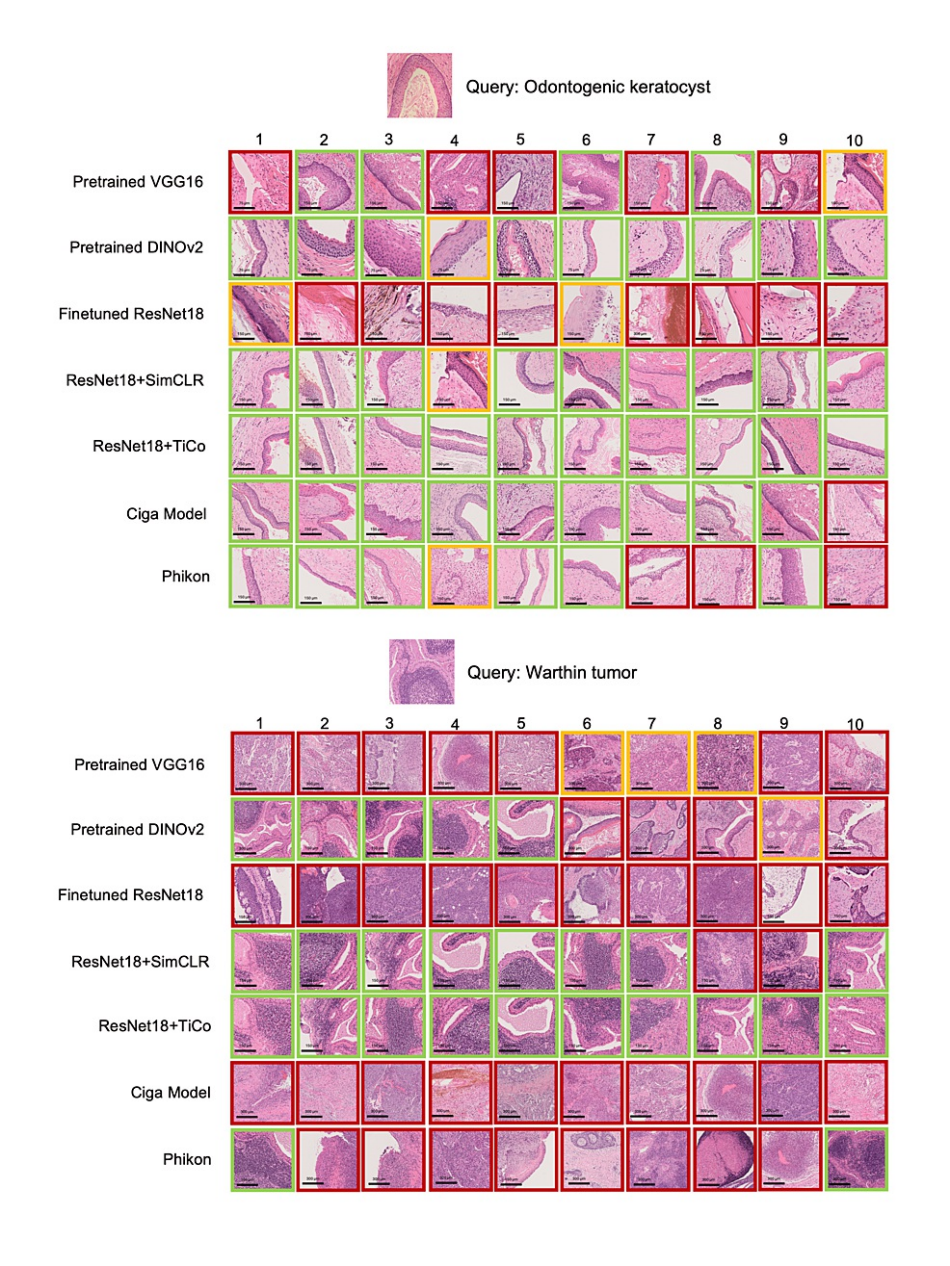
Comparison of the top 10 results of all models for out-of-domain-phonecam queries from different categories. ResNet18+SimCLR and ResNet18+TiCo are consistent with the result that provides the highest Acc for different query categories. More examples are presented in Figure A5 in the Appendices. Green outline: accurate diagnosis category; yellow outline: differential diagnosis categories; red outline: inaccurate diagnosis category.

The accuracy calculation excluded the histologic similarity between the query and retrieved images, which provides additional information about the histologic features during diagnosis. To verify whether the SSL models retrieved histologically similar images despite the low Mean-Acc, Mean-HI was introduced. Mean-HI excludes accurate diagnosis and differential diagnosis categories, which are similar to the query and include other inaccurate categories. The lowest overall inaccuracy was consistently achieved by the SSL models, except for the high-magnification in-domain queries, indicating that these models are best at retrieving the most histologically similar images beyond accurate diagnosis (Figures 3C, 3D and Table A5 in the Appendices). The top 10 retrieved images of representative cases found by all the tested models are shown in Figure 4 and Figure A5 in the Appendices.

## Discussion

The diagnosis of oral pathology has long depended on histopathological image observation, which can be a burden for pathologists, especially when dealing with rare cases. In the last decade, various machine learning methods, such as supervised learning methods for classification, detection, and segmentation, have been proposed to aid in clinical and histopathological diagnosis and to improve speed and accuracy to avoid delays in diagnosis [3,4,9-12,26]. However, the exploration for oral histopathology diagnosis has been hampered by the difficulty of obtaining an adequately extensive database that includes rare cases and constructing an effective model. To our knowledge, this is the first study to construct a large database of 30 oral tumor categories, with an additional 21 categories used as the model-training dataset.

Many have argued that CBIR has greater advantages in this field. Pathologists can review CBIR results to make a final decision. However, decision bias may occur when the algorithm is unreliable. To find the best way to represent images for CBIR, we compared different methods of training the feature encoder. We then ranked the similarity of all images in the database to the test queries. The gradual concept of similarity and the multiple-ranked results of CBIR pose a challenge in interpretation. Previous research on CBIR for histopathology images uses the number of accurate image categories retrieved or the majority of categories retrieved at the top-k [10,27]. Here, four evaluation measures were used to better capture the CBIR performance on different aspects: Mean-AUC from the whole database similarity rank; Mean-Acc, %query, and Mean-HI from the top 10 most similar images. Mean-AUC assumes all rank cut-points are relevant to model performance in extracting histologic features, while the top 10 similar results are relevant during image observation by pathologists in future CBIR implementation. Our findings suggest that model training for feature extraction using an in-house dataset with SSL methods outperforms other popular methods in retrieving images with an accurate diagnosis and similar histology: in-domain queries in 73% of categories and out-of-domain-phonecam queries in 64% of categories (Table 1). This was supported by their Mean-Acc, which was superior in 63% of in-domain query categories and 82% of out-of-domain-phonecam query categories (Table A3 in the Appendices). There was no significant difference in Mean-Acc between the SimCLR and TiCo models for both query categories. The Mean-Acc superiority of the SSL models was consistent at the in-domain low and moderate magnification levels (Figure 3A) and at out-of-domain-phonecam at all magnification levels (Figure 3B). Additionally, both SSL models retrieved fewer images without histologic similarity at all magnification levels for both query categories (Figures 3C, 3D), meaning when low accuracy is achieved in the result, the users get several options that are histologically similar upon CBIR implementation with the SSL models because they belong to the textbook differential diagnosis categories. From this result, users could proceed with the additional tests more easily than having to do the preliminary reference search manually. Our dataset had overlapping image patches with similar histologic features. In this situation, the SSL method was superior because it compensated for the lack of a labeled dataset for learning representations that cluster the data during training based on semantic classes in conjunction with convolutional neural networks as feature extractors, regardless of the category [15].

The most impressive performance was shown for Warthin’s tumor query, with Mean-AUC values greater than 0.960 in every query set using the SSL models (Table 1). One possible reason is that Warthin’s tumors consist of varying proportions of papillary cystic structures lined by two layers of oncocytic epithelial cells and a lymphoid stroma with germinal centers. It is one of the most common tumors of the salivary gland, especially the parotid gland, and is generally easy to diagnose microscopically owing to its characteristic pattern [18].

The SLL models were successful for most of the test query categories. Out of those categories, the Mean-Acc of the SLL model for the ameloblastoma query was lower (Table A1 in the Appendices). Although ameloblastoma is one of the most common odontogenic tumors, it has diverse histologic variants: follicular, plexiform, acanthomatous, granular, basaloid, desmoplastic, or a mixture of these [16]. This diversity requires an adequate representation of each subtype in the database for greater accuracy. However, the %query indicated that the models retrieved a similar ameloblastoma type in the top 10 for more than 93% of the queries tested (Table A4 in the Appendices). With several differential diagnoses of ameloblastoma included in the database, the best Mean-HI obtained for the in-domain ameloblastoma query was 5.98 by SimCLR (Table A5 in the Appendices). These categories can be considered histologically similar only if the characteristics of certain subtypes are captured. For example, islands of odontogenic epithelium with ameloblastic features in the follicular type may resemble ameloblastic fibroma [18]. Updating the database with newly encountered subtypes continuously would improve the accuracy of rare tumor subtypes.

Although CBIR works by retrieving similar images that can be considered a digital second opinion, the result may contain images from different categories, with many having similar or indistinguishable histology. Arguably, the range of Mean-Acc values obtained with the SSL models, i.e., 1.00 to 8.94 (Table 1 and Table A3 in the Appendices), is considerably wide. However, 55% to 100% of the total queries retrieved at least one of 10 images from the correct category (Table A4 in the Appendices), and 6.46 to 1.12 out of the 10 images had no histologic similarity to the query image (Table A5 in the Appendices). This implies that displaying the complete top 10 results, including the correct diagnosis and differential diagnosis, as shown in Figure 4 and Figure A5 in the Appendices, could be significant for pathologists to narrow the differential diagnoses and conduct further research efficiently. To further improve usability, it is necessary to include clinical and other findings, such as the location of the tumor, patient history, and diagnostic criteria, which are usually essential to making a diagnosis by pathologists, when developing a CBIR system, especially in the oral region where tissue types are diverse.

This study implements patch-based CBIR. Some CBIR systems can analyze WSIs, of which implementation is prospective in developed countries. As expensive WSI scanners are not universally installed in oral laboratories, the image-capturing equipment accessible to pathologists differs considerably across regions. Microscope images captured directly using a smartphone camera could be the easiest mode for education, image sharing, and case consultation [28,29]. By using patch-based CBIR where pathologists only need to select the tumor areas and capture them with smartphone cameras to create input, this technology is more accessible globally. Variations in image color and resolution resulting from these differences hinder obtaining reliable results. We tested the robustness of each model to domain shifts by testing the models on out-of-domain queries using WSIs from multiple institutions captured by different scanners and smartphone cameras. SSL models performed best for most query categories, with SimCLR or TiCo achieving the best Mean-AUC for over 68% of out-of-domain query categories, from 0.839 to 0.999 (Table 1), confirming the previous finding that SSL is more robust to domain shifts than supervised learning in some datasets, including pathological images [30]. Interestingly, the performances of the vision transformer models (pre-trained DINOv2 and Phikon) always come second best to the SSL models in Mean-AUC and are comparable in Mean-Acc to that of the SSL models on out-of-domain query sets (Table 1 and Table A3 in the Appendices). Although further investigation is needed, this result may be considered when choosing methods for a CBIR system. If the system is designed for an in-house database and query or a scenario applicable in large hospitals, SSL models trained on in-house cases are the optimal choice. However, where the system is designed to handle out-of-domain queries, using a pre-trained vision transformer model becomes a viable alternative to eliminate the need to train the SSL model, which could be computationally expensive.

Limitations of this study include that the experiments involved a single query to retrieve similar images. An algorithm that supports more information in the query, such as multiple query algorithms and filters for location or other diagnostic criteria, would improve retrieval accuracy and provide better support for diagnosis. Our study is limited to test queries from the same geographical area as the SSL model-training dataset. Collecting query cases from a more diverse area would be beneficial in future CBIR development to further challenge the generalizability of the result. The comparative methodology did not emphasize histopathology characteristics that differentiate between benign or malignant tumors, such as capsule invasion and mitotic activity in basal cell adenoma vs. basal cell adenocarcinoma but focused on how such image retrieval tools would be beneficial in reducing several differential diagnoses and recalling diagnosis criteria before following up with ancillary tests if necessary. Image retrieval is less likely to mislead decision-makers owing to model overfit than a conventional classification method that predicts the possible tumor diagnosis. Nonetheless, a sequel of observations would still be needed when image retrieval is utilized. This study provides insights as the first step to developing a CBIR algorithm by observing retrieval accuracy with strict tumor category criteria on a relatively small database and did not investigate the impact of the result on decision-making in clinical settings. The implementation of CBIR as a well-rounded system to be incorporated into the comprehensive diagnostic process is beyond the scope of this study and observation of the interaction between pathologists and a CBIR system for common and rare diagnoses is needed before the system is used in clinical settings.

## Conclusions

This study highlighted various methods to develop an effective CBIR model and proposed four key measures to determine the best approach for future clinical usage. These measures capture different aspects of CBIR performance that would be relevant for diagnosis decisions in clinical settings. We have shown that using SSL methods trained on an oral tumor dataset is an effective way to develop a CBIR system for the histopathological diagnosis of oral tumors compared to other commonly used methods. Vision transformer models trained on a large image dataset, though slightly less effective than SSL models, still provide strong performance and could be a viable alternative for out-of-domain queries. These approaches have considerable potential to create a clinically useful image retrieval system that accelerates the diagnostic process and improves accuracy.

## Figures and Tables

**Figure 5:**
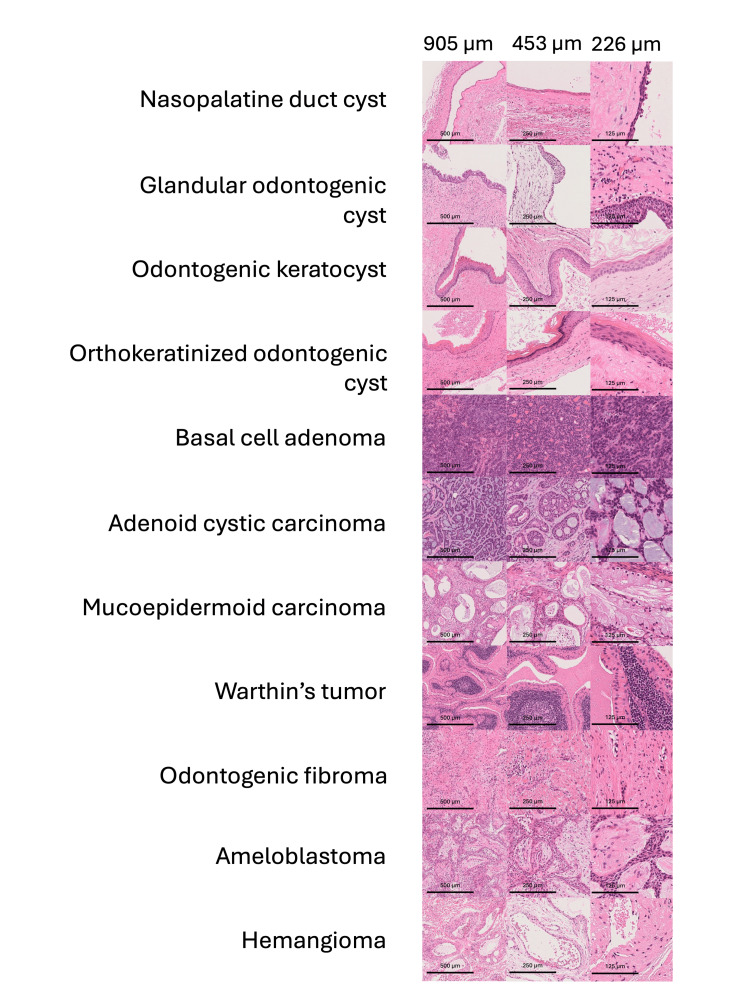
Examples of set-A query images from each category for each magnification level. The total number of image patches extracted from whole-slide images is 1,320 images.

**Figure 6:**
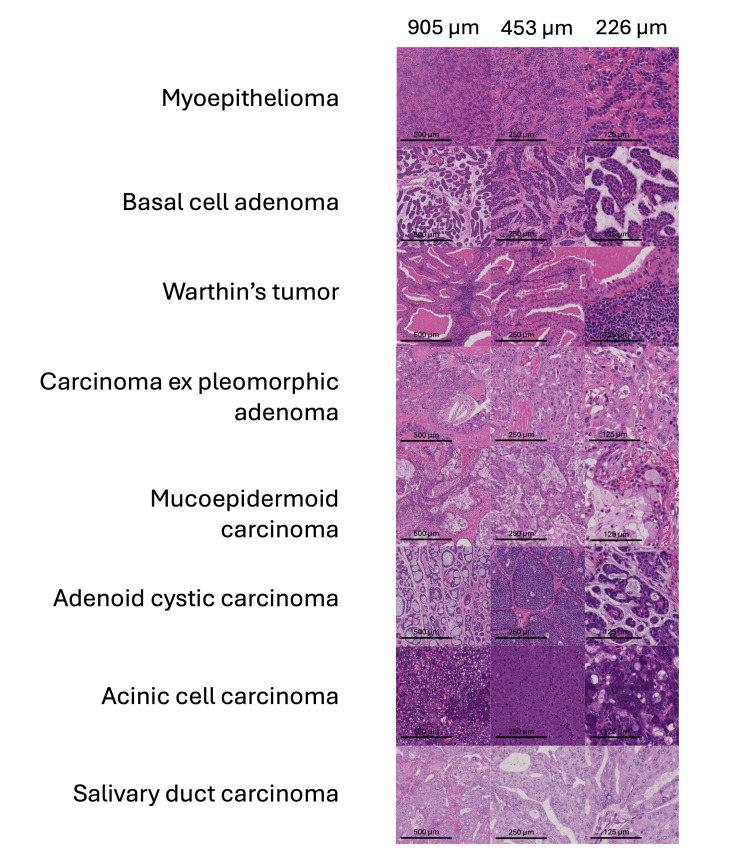
Examples of set-B query images from each category for each magnification level. The total number of image patches extracted from the whole-slide images is 660 images.

**Figure 7:**
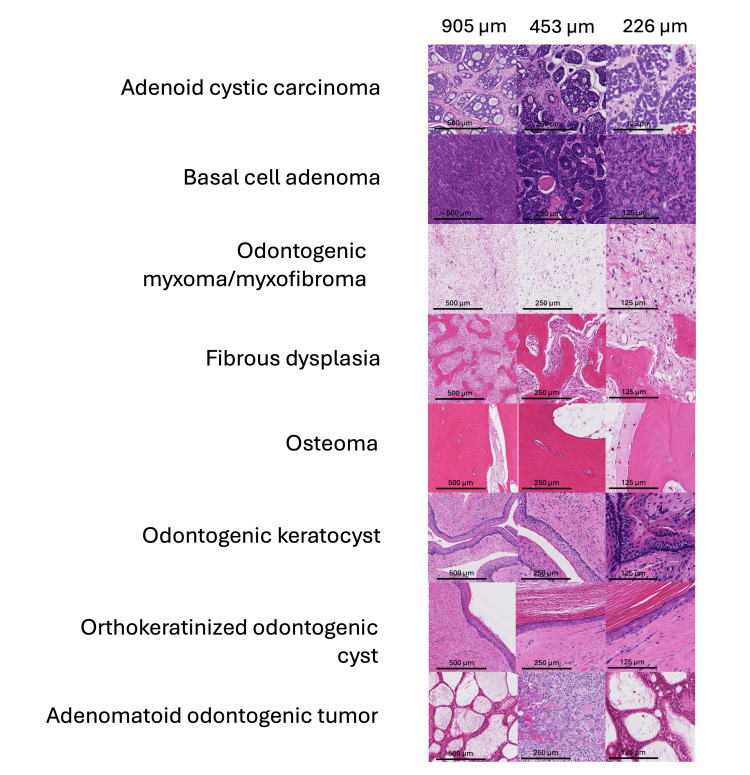
Examples of set-C query images from each category for each magnification level. The total number of image patches extracted from the whole-slide images is 1,200 images.

**Figure 8:**
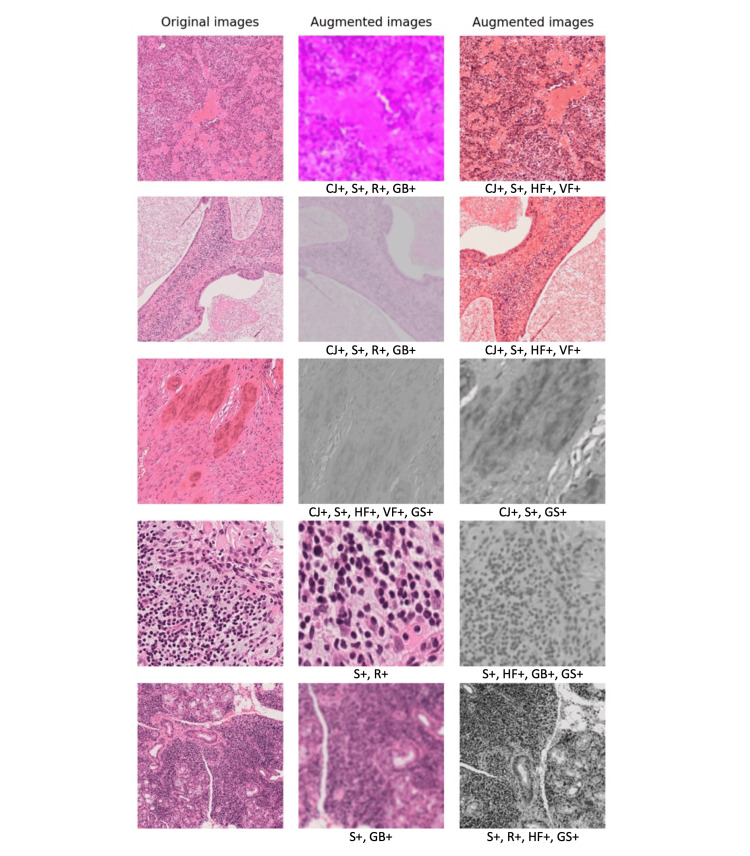
Data augmentation examples. Random color jittering (CJ+), grayscale (GS+), image scaling (S+), horizontal and vertical flips (HF+ and VF+), 90-degree rotation (R+), and Gaussian blur (GB+) were used during self-supervised learning (SSL) training. Color normalization was used during model training but is not included in this visualization.

**Figure 9:**
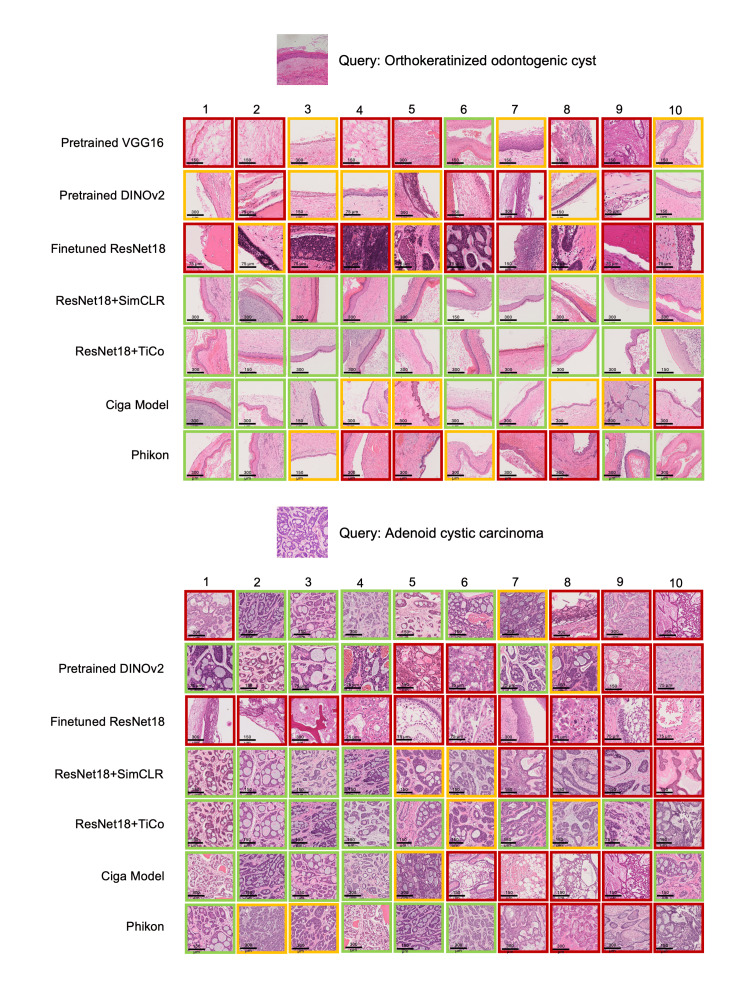
Further examples of comparisons of the top 10 results of all models for out-of-domain-phonecam queries from different categories. ResNet18+SimCLR and ResNet18+TiCo are consistent with the result providing the highest Acc for different query categories. The categories of retrieved images belonging to the differential diagnoses show which retrieved images have histologic similarity to the query. This comparison demonstrates the SSL model’s capability to retrieve histologically similar images when the exact accurate diagnosis is not retrieved. The pre-trained DINOv2 and Phikon also show such potential albeit less consistently across the query category than the self-supervised learning (SSL) models. Green outline: accurate diagnosis category; yellow outline: differential diagnosis categories; red outline: inaccurate diagnosis categories.

## Appendices

Method 1: Dataset image collection, database construction, and test query preparation

Dataset

We collected diagnostic slides of the oral tumor categories described in chapters 7 and 8 of the WHO Classification of Head and Neck Tumours, 4th Edition [16]. Patients were diagnosed in 2001-2022 and underwent surgery at Tokyo Medical and Dental University (TMDU) Hospital. Patients or their surrogates had the option to withdraw from this study through public notices according to the approved protocols. This study was approved by the Institutional Review Board (IRB) of TMDU (No. D2019-087). Some slides that were lost, broken, or required diagnostic confirmation with immunohistochemistry (IHC) staining were remade from the paraffin-embedded tissue blocks. Additional IHC staining was done for the secretory carcinoma and the atypical acinic cell carcinoma cases older than 2017. Categories with fewer than five cases were excluded. All slides that fulfill the inclusion criteria were scanned using a NanoZoomer S210 slide scanner (C13239-01; Hamamatsu Photonics, Shizuoka, Japan) at 40× magnification. The tumor areas were annotated by a pathology resident and verified by board-certified pathologists. We included the tumor areas that are typical to the tumor category while excluding the normal tissue and the severe artifacts such as torn or folded tissue. Image patches were then randomly extracted from the annotated tumor areas with three different sizes: 905 µm, 453 µm, and 226 µm. Twenty image patches were extracted with each magnification. The use of three different sizes was to accommodate different magnification levels and preserve the histologic information at the tissue and cellular level as much as possible. The dataset comprises 49,243 image patches from 51 categories, covering approximately 50% of the oral tumor categories (Table A1).

Database Construction

A database from a subset of the dataset containing at least 10 cases was compiled. Image representations from each model’s encoder were stored in the database (Figure 2A). It contains 33,356 image patches from 30 oral tumor categories (Table A1).

Test Queries

The test queries were the cases available in the hospital repository after the collection of the database case was finished and were representatives of the tumor major categories in the database: odontogenic cysts, odontogenic tumors, benign and malignant tumors of the salivary gland, maxillofacial bone tumor, and soft tissue tumor. Slides that include severe artifacts that are impossible to avoid when extracting image patches were excluded. We prepared three query sets from different hospitals to test the performance: query case set-A was collected between 2022 and 2023 from the repository of the same institution (TMDU) as the database. Only the tumor categories that have two representative cases were included as the test queries. Finally, 11 tumor categories with two cases in each were used as the test queries. Histopathologic slides were scanned to create WSIs for in-domain queries with the same device as the database image. Three selected tumor areas that are typical of the tumor type from the same slides were photographed with smartphone cameras (Samsung S21FE: 12 megapixels; iPhone 6: 8 megapixels; and Motorola g8: 16 megapixels) using an Olympus BX53 microscope with 10×, 20×, and 40× objective lens magnification. All smartphone images were cropped to square, removing the outer rim of the microscope lens, to create out-of-domain-phonecam queries. The location (indoor laboratory environment) and the amount of light from the microscope were unchanged when taking the smartphone images. The query case set B was compiled from the University of Tokyo case (approved by the IRB of The University of Tokyo; No. 2019158NI). Eleven cases from eight salivary gland tumor categories were included in this study. Only benign and malignant tumors of the salivary gland could be collected for out-of-domain-B since no other major categories exist in the repository. Histopathologic slides were scanned using a NanoZoomer 2.0HT slide scanner (C9600-12, Hamamatsu Photonics) to create WSIs for out-of-domain-B queries. The query case set C was collected from Teikyo University Hospital from 2018 to 2023 (approved by the IRB of Teikyo University; No. 23-054). Only the tumor categories that have two representative cases were included as the test queries. Twenty cases from eight oral tumor categories were included in the study. The histopathologic slides were scanned using a NanoZoomer XR slide scanner (C12000-02; Hamamatsu Photonics) to create WSIs for out-of-domain-C queries. Patients for query cases set-B and set-C or their surrogates had the option to withdraw from this study through public disclosure according to the approved protocols.

For the WSI queries, the same method was used to create image patches from the WSI as from the database image. All scanned WSIs were annotated to three to four representative tumor areas per WSI. From these areas, 20 image patches per magnification level (905 µm, 453 µm, and 226 µm) per slide were extracted to create image patches. The total number of image patches used for the evaluation was 2,520 images from the WSI and 594 smartphone images. The number of images analyzed in each category is detailed in Tables A3-A5.

The representation of each query image was calculated with each tested model. The nearest-neighbor search was performed based on cosine similarity with the database images. Examples of query images for each category in each set can be found in Figures A1-A3. The detailed methods for database construction, including the tumor areas selection, patch extraction, feature extraction code, and image retrieval were adapted from our previous study [17].

Method 2: Model preparation and training

ImageNet-1k Pre-trained CNN

We tested VGG16 pre-trained on 1.2 million images from ImageNet after it was shown to perform well as an image classifier in several studies [21,22]. In this study, the block4_conv3 layer was used as the feature extractor because the middle layer of a convolutional neural network (CNN) architecture has been shown to capture features that are more suitable for histopathology images [11].

ImageNet-22k Pre-trained Vision Transformer

We used the DINOv2 ViT-L/14 model pre-trained with the SSL method on general images from ImageNet-22k [25]. The last layer was used for feature extraction. The images were cropped to 252 pixels owing to the input size restrictions.

Fine-Tuned CNN

We fine-tuned all the ImageNet-1k pre-trained ResNet18 models on our dataset using the supervised learning method to classify 51 categories. All the layers were trained with a learning rate of 0.001, a batch size of 32, and 100 epochs in PyTorch 1.11.0 (Meta AI, Astor Place, New York). During training, a random 90-degree rotation, random horizontal and vertical flips, color jitter, Gaussian blur, and color normalization transformation were performed. The training-to-test ratio was 8:2.

CNN Trained With SSL Methods

Contrastive (SimCLR [20]) and noncontrastive methods (TiCo [24]) were investigated. ResNet18 is used as the backbone. During training, random color jittering, grayscale, image scaling, horizontal and vertical flipping, 90-degree rotation, Gaussian blurring, and color augmentation were implemented. Both models were trained with Lightly version 1.3.3, with a learning rate of 1.2, a batch size of 32 × 32 (with accumulated gradients), and 1,000 epochs. Examples of original and augmented images are shown in Figure A4. The ResNet18 backbone model trained on 57 histopathology image datasets (38,594 image patches and 24,923 WSIs) developed by Ciga et al. (2022) was also used for comparison [13]. The code for SSL model training is available at "https://github.com/rannyrh/oralpath_CBIR".

Histopathology Image-Trained Vision Transformer

A vision transformer-based model, Phikon, was trained with 40 million pan-cancer tiles extracted from The Cancer Genomic Atlas (TCGA) using the masked image modeling (MIM) method as an SSL framework. MIM learns meaningful representation by randomly masks portions of an image and trying to reconstruct those masked portions. This model was developed by Owkin Inc. (Paris, France) [23].

Figure A1

Figure A2

Figure A3

Figure A4

Figure A5

Table A1

**Table 2:** The tumor categories included in the dataset, their corresponding ICD-O codes, and the total number of cases. A total of 51 categories are compiled as the image dataset. Some categories do not correspond to the ICD-O but are described in the WHO Classification of Head and Neck Tumours, 4th Edition. Thirty categories consisting of 10 to 20 cases, marked in *italics*, were included in the CBIR database. ICD-O: International Classification of Diseases for Oncology; CBIR: content-based image retrieval; NOS: not otherwise specified; MALT: mucosa-associated lymphoid tissue.

Diagnosis	ICD-O code	Total case
Pleomorphic adenoma	8940/0	27
Radicular cyst	-	21
Dentigerous cyst	-	21
Odontogenic keratocyst	-	17
Salivary duct carcinoma	8500/3	15
Acinic cell carcinoma	8550/3	15
Ossifying fibroma	9262/0	15
Lipoma	8850/0	14
Ameloblastoma	9310/0	14
Adenoid cystic carcinoma	9310/0	13
Mucoepidermoid carcinoma	8430/3	13
Fibrous dysplasia	-	13
Adenocarcinoma, NOS	8140/3	12
Myoepithelioma	8982/0	12
Odontogenic fibroma	9321/0	12
Cemento-osseous dysplasia	-	12
Nasopalatine duct cyst	-	12
Hemangioma	9120/0	11
Calcifying odontogenic cyst	9301/0	11
Odontogenic myxoma/myxofibroma	9320/0	11
Ameloblastic fibroma	9330/0	11
Glandular odontogenic cyst	-	11
Inflammatory collateral cyst	-	11
Orthokeratinized odontogenic cyst	-	11
Basal cell adenoma	8147/0	10
Basal cell adenocarcinoma	8147/3	10
Warthin’s tumor	8561/0	10
Carcinoma ex pleomorphic adenoma	8941/3	10
Osteoma	9180/0	10
Osteosarcoma, NOS	9180/3	10
Aneurysmal bone cyst	9260/0	10
Cemento-ossifying fibroma	9274/0	10
Adenomatoid odontogenic tumor	9300/0	10
MALT lymphoma	9699/3	9
Ameloblastoma, unicystic type	9310/0	9
Lateral periodontal cyst	-	9
Simple bone cyst	-	8
Cystadenoma	8440/0	8
Epithelial/myoepithelial carcinoma	8562/3	7
Complex odontoma	9282/0	7
Polymorphous adenocarcinoma	8525/3	7
Secretory carcinoma	8502/3	6
Ameloblastoma, extraosseous/peripheral type	-	6
Compound odontoma	9281/0	5
Odontoma	9280/0	5
Oncocytoma	8290/0	5
Cementoblastoma	9273/0	5
Clear cell carcinoma	8310/3	5
Primary intraosseous carcinoma, NOS	9270/3	5
Ductal papilloma	8503/0	5
Myoepithelial carcinoma	8982/3	5

Table A2

**Table 3:** List of test query categories and all their respective differential diagnoses that are represented in the database for Mean-HI evaluation. NOS: not otherwise specified; HI: histologic inaccuracy.

Query category	Differential diagnoses		
Acinic cell carcinoma	Salivary duct carcinoma	Secretory carcinoma	Mucoepidermoid carcinoma
Adenoid cystic carcinoma	Basal cell adenocarcinoma	Basal cell adenoma	
Adenomatoid odontogenic tumor	Ameloblastoma	Mucoepidermoid carcinoma	
Ameloblastoma	Adenomatoid odontogenic tumor	Ameloblastic fibroma	
Basal cell adenoma	Adenoid cystic carcinoma	Basal cell adenocarcinoma	Myoepithelioma
Carcinoma ex pleomorphic adenoma	Salivary duct carcinoma	Adenocarcinoma, NOS	Mucoepidermoid carcinoma
Fibrous dysplasia	Ossifying fibroma	Osteosarcoma, NOS	
Glandular odontogenic cyst	Nasopalatine duct cyst	Inflammatory collateral cyst	Mucoepidermoid carcinoma
Hemangioma	Ossifying fibroma	Aneurysmal bone cyst	
Mucoepidermoid carcinoma	Adenocarcinoma, NOS	Warthin’s tumor	Carcinoma ex pleomorphic adenoma
Myoepithelioma	Mucoepidermoid carcinoma	Basal cell adenoma	
Nasopalatine duct cyst	Glandular odontogenic cyst	Inflammatory collateral cyst	
Odontogenic fibroma	Odontogenic myxoma/myxofibroma	Ossifying fibroma	
Odontogenic keratocyst	Orthokeratinized odontogenic cyst	Ameloblastoma	
Odontogenic myxoma/myxofibroma	Odontogenic fibroma		
Orthokeratinized odontogenic cyst	Odontogenic keratocyst	Ameloblastoma	
Osteoma	Ossifying fibroma	Fibrous dysplasia	
Salivary duct carcinoma	Acinic cell carcinoma	Secretory carcinoma	Adenocarcinoma, NOS
Warthin’s tumor	Mucoepidermoid carcinoma		

Table A3

**Table 4:** Mean-Acc (SD) of each test query category in in- and out-of-domain image queries. The performances of the SSL models are superior for most categories, further validating the robustness of the models under a wide range of histopathological image conditions. The highest Mean-Acc for each category is marked in *italics*. SSL: self-supervised learning; Acc: accuracies.

Query	Category	Pre-trained VGG16	Pre-trained DINOv2	Fine-tuned Resnet18	ResNet18 + SimCLR	ResNet18 + TiCo	Ciga model	Phikon
In-domain (n = 120 images from 2 cases)	Nasopalatine duct cyst	1.38 (0.28)	1.48 (0.44)	1.53 (1.02)	2.59 (0.94)	3.48 (0.82)	1.93 (0.43)	2.38 (0.66)
	Glandular odontogenic cyst	2.55 (1.00)	2.70 (0.65)	1.72 (0.52)	3.13 (0.77)	3.22 (0.71)	2.07 (0.73)	2.58 (0.65)
	Odontogenic keratocyst	2.41 (0.53)	4.96 (0.33)	5.39 (0.50)	6.79 (0.92)	6.73 (0.76)	2.79 (0.82)	7.13 (0.78)
	Orthokeratinized	1.68 (0.96)	3.94 (1.49)	3.04 (1.83)	6.19 (1.84)	6.83 (1.93)	2.38 (1.01)	4.92 (1.37)
	odontogenic cyst							
	Basal cell adenoma	5.25 (0.85)	3.43 (1.51)	6.11 (0.44)	5.13 (1.34)	5.61 (1.72)	4.34 (0.58)	5.73 (1.14)
	Adenoid cystic carcinoma	2.86 (1.10)	3.28 (0.66)	3.15 (1.37)	4.09 (0.99)	4.36 (0.98)	2.14 (1.13)	3.13 (1.02)
	Mucoepidermoid carcinoma	3.10 (0.69)	2.78 (0.89)	1.33 (0.26)	2.87 (0.83)	2.89 (1.10)	1.85 (0.94)	3.71 (0.75)
	Warthin’s tumor	6.13 (1.32)	5.93 (2.65)	7.63 (0.88)	8.94 (0.56)	8.38 (1.05)	4.97 (1.16)	8.23 (1.10)
	Odontogenic fibroma	2.05 (1.36)	2.45 (0.89)	0.99 (0.32)	2.03 (1.33)	2.04 (1.40)	1.29 (0.75)	1.74 (1.01)
	Ameloblastoma	1.67 (0.79)	1.83 (0.80)	1.41 (0.65)	2.73 (1.19)	2.42 (0.88)	1.09 (0.32)	1.71 (0.69)
	Hemangioma	3.20 (1.04)	2.51 (1.07)	3.03 (1.23)	5.39 (1.06)	5.11 (1.15)	2.93 (1.07)	4.56 (1.60)
	Average	3.20 (1.50)	3.21 (1.32)	3.21 (2.22)	4.53 (2.16)	4.64 (2.06)	2.53 (1.19)	4.16 (2.29)
Out-of-domain-phonecam (n = 54 images from 2 cases)	Nasopalatine duct cyst	0.98 (0.59)	1.85 (0.44)	0.87 (0.39)	2.15 (0.40)	2.52 (0.21)	1.06 (0.40)	1.57 (0.59)
	Glandular odontogenic cyst	1.91 (0.86)	2.48 (0.77)	2.00 (0.54)	3.28 (0.99)	2.98 (1.10)	2.00 (0.33)	2.91 (0.64)
	Odontogenic keratocyst	2.83 (0.63)	4.24 (1.34)	0.31 (0.56)	5.19 (0.76)	5.52 (0.81)	3.41 (1.16)	3.93 (0.98)
	Orthokeratinized odontogenic cyst	1.87 (1.04)	2.67 (0.40)	1.67 (1.25)	3.78 (1.68)	3.59 (1.86)	2.20 (0.82)	2.72 (0.42)
	Basal cell adenoma	2.00 (0.39)	2.15 (0.43)	0.19 (0.25)	2.48 (0.97)	2.43 (1.00)	1.19 (0.62)	2.24 (1.10)
	Adenoid cystic carcinoma	2.00 (1.20)	2.67 (0.96)	0.74 (0.62)	2.91 (0.98)	2.87 (1.42)	1.69 (1.33)	1.50 (1.27)
	Mucoepidermoid carcinoma	2.43 (0.67)	1.93 (0.76)	0.31 (0.33)	1.93 (0.52)	1.78 (1.00)	0.87 (0.70)	0.89 (0.70)
	Warthin’s tumor	3.17 (1.80)	5.06 (2.33)	1.26 (0.89)	7.28 (2.31)	7.26 (1.46)	2.83 (1.69)	2.31 (1.45)
	Odontogenic fibroma	0.56 (0.22)	0.91 (0.22)	0.04 (0.09)	1.09 (0.59)	1.33 (0.79)	1.44 (0.37)	0.70 (0.30)
	Ameloblastoma	1.15 (0.85)	2.02 (0.37)	1.22 (0.34)	3.26 (1.25)	2.76 (0.88)	1.28 (0.44)	2.02 (0.59)
	Hemangioma	2.39 (0.30)	1.37 (0.81)	0.26 (0.24)	3.35 (0.65)	3.43 (0.62)	1.98 (0.84)	2.00 (1.08)
	Average	1.93 (1.11)	2.48 (1.47)	0.81 (0.83)	3.33 (1.93)	3.31 (1.92)	1.81 (1.11)	2.07 (1.21)
Out-of-domain-B	Myoepithelioma (n = 60 images from 1 case)	0.88 (0.29)	1.97 (0.34)	1.27 (0.89)	1.00 (0.26)	1.17 (0.26)	1.38 (0.48)	1.73 (0.33)
	Basal cell adenoma (n = 60 images from 1 case)	2.62 (0.35)	1.37 (0.40)	2.92 (0.75)	2.42 (0.18)	1.98 (0.20)	1.62 (0.40)	3.03 (0.56)
	Warthin’s tumor (n = 60 images from 1 case)	5.70 (0.96)	7.63 (0.71)	2.30 (2.46)	8.88 (0.26)	8.23 (0.33)	2.52 (0.13)	7.28 (0.87)
	Carcinoma ex pleomorphic adenoma (n = 60 images from 1 case)	1.45 (0.00)	1.48 (0.78)	1.15 (0.64)	3.33 (0.53)	3.07 (0.18)	1.52 (0.21)	1.72 (0.29)
	Mucoepidermoid carcinoma (n = 120 images from 2 cases)	2.09 (1.02)	2.14 (0.53)	0.62 (0.51)	1.61 (0.84)	1.73 (0.87)	1.90 (0.90)	1.61 (1.12)
	Adenoid cystic carcinoma (n = 120 images from 2 cases)	3.35 (1.32)	4.15 (1.24)	1.82 (0.91)	3.31 (2.39)	3.23 (2.02)	2.55 (1.79)	3.88 (1.76)
	Acinic cell carcinoma (n = 120 images from 2 cases)	3.99 (2.37)	5.00 (1.76)	2.43 (0.91)	4.95 (1.90)	5.63 (2.38)	3.53 (2.51)	5.78 (1.41)
	Salivary duct carcinoma (n = 60 images from 1 case)	3.95 (0.36)	4.12 (1.68)	0.90 (0.74)	5.05 (0.26)	4.68 (1.07)	4.23 (3.38)	5.40 (2.04)
	Average	3.04 (1.78)	3.56 (2.13)	1.66 (1.19)	3.68 (2.50)	3.67 (2.47)	2.47 (1.78)	3.79 (2.33)
Out-of-domain-C	Adenoid cystic carcinoma (n = 180 images from 3 cases)	3.39 (1.06)	3.04 (1.52)	1.44 (0.58)	2.74 (1.34)	3.04 (0.95)	1.85 (0.64)	3.62 (0.56)
	Basal cell adenoma (n = 180 images from 3 cases)	3.94 (1.45)	2.35 (0.85)	0.95 (0.55)	2.23 (1.90)	2.75 (2.11)	1.19 (0.56)	3.01 (1.63)
	Odontogenic myxoma/myxofibroma (n = 180 images from 3 cases)	4.37 (2.25)	6.00 (1.66)	0.96 (0.43)	5.62 (1.48)	6.05 (1.64)	5.20 (1.60)	4.42 (1.70)
	Fibrous dysplasia (n = 120 images from 2 cases)	2.37 (1.22)	2.90 (0.59)	0.48 (0.19)	2.07 (1.03)	1.48 (0.79)	1.52 (0.99)	1.45 (1.10)
	Osteoma (n = 180 images from 3 cases)	4.22 (1.52)	4.63 (1.39)	1.97 (0.19)	4.27 (2.18)	4.66 (0.86)	3.93 (0.74)	4.93 (1.65)
	Odontogenic keratocyst (n = 120 images from 2 cases)	2.65 (1.62)	3.99 (1.26)	0.28 (0.56)	3.09 (3.27)	3.13 (3.16)	3.56 (1.87)	3.38 (2.05)
	Orthokeratinized odontogenic cyst (n = 120 images from 2 cases)	1.53 (0.98)	3.60 (1.39)	0.02 (0.04)	3.95 (2.32)	4.06 (2.07)	2.15 (1.30)	3.59 (2.31)
	Adenomatoid odontogenic tumor (n = 120 images from 2 cases)	1.79 (0.42)	1.90 (0.62)	2.19 (0.89)	3.07 (0.61)	2.85 (0.77)	0.90 (0.64)	2.37 (1.35)
	Average	3.22 (1.71)	3.64 (1.76)	1.52 (1.36)	3.45 (2.13)	3.63 (2.08)	2.64 (1.81)	3.47 (1.81)

Table A4

**Table 5:** Percentage of queries that retrieved at least one correct diagnosis among the top 10 results for each test query category (%query) in in- and out-of-domain image queries. The performance of the SSL models was superior in most categories. The highest %query for each category is marked in *italics* (%). SSL: self-supervised learning.

Query	Category	Pre-trained VGG16	Pre-trained DINOv2	Fine-tuned ResNet18	ResNet18 + SimCLR	ResNet18 + TiCo	Ciga model	Phikon
In-domain (n = 120 images from 2 cases)	Nasopalatine duct cyst	80.00	80.00	71.67	93.33	96.67	87.50	95.83
	Glandular odontogenic cyst	92.50	95.00	93.33	99.17	99.17	91.67	95.00
	Odontogenic keratocyst	88.33	100.00	100.00	100.00	96.67	88.33	100.00
	Orthokeratinized odontogenic cyst	84.17	96.67	91.67	98.33	100.00	92.50	100.00
	Basal cell adenoma	100.00	96.67	100.00	100.00	100.00	100.00	100.00
	Adenoid cystic carcinoma	94.17	96.67	98.33	100.00	100.00	81.67	100.00
	Mucoepidermoid carcinoma	94.17	88.33	94.17	98.33	97.50	80.00	98.33
	Warthin’s tumor	99.17	97.50	100.00	100.00	99.17	95.83	98.33
	Odontogenic fibroma	85.83	94.17	70.83	73.33	71.67	68.33	85.00
	Ameloblastoma	84.17	81.67	80.00	92.50	93.33	70.00	89.17
	Hemangioma	91.67	92.50	98.33	100.00	100.00	93.33	100.00
	Average	90.38	92.65	90.76	95.91	95.83	86.29	96.52
Out-domain-phonecam (n = 54 images from 2 cases)	Nasopalatine duct cyst	53.70	92.59	68.52	98.15	100.00	74.07	87.04
	Glandular odontogenic cyst	88.89	98.15	81.48	100.00	100.00	96.30	100.00
	Odontogenic keratocyst	90.74	88.89	16.67	98.15	100.00	96.30	92.59
	Orthokeratinized odontogenic cyst	75.93	92.59	62.96	88.89	88.89	88.89	96.30
	Basal cell adenoma	96.30	90.74	12.96	85.19	88.89	75.93	83.33
	Adenoid cystic carcinoma	85.19	85.19	42.59	88.89	74.07	64.81	64.81
	Mucoepidermoid carcinoma	94.44	81.48	25.93	94.44	70.37	61.11	53.70
	Warthin’s tumor	72.22	98.15	51.85	96.30	98.15	72.22	75.93
	Odontogenic fibroma	51.85	55.56	3.70	79.63	79.63	90.74	66.67
	Ameloblastoma	61.11	83.33	88.89	94.44	98.15	79.63	88.89
	Hemangioma	94.44	66.67	24.07	81.48	90.74	81.48	85.19
	Average	78.62	84.85	43.60	91.41	89.90	80.13	81.31
Out-of-domain-B	Myoepithelioma (n = 60 images from 1 case)	43.33	93.33	63.33	55.00	56.67	75.00	78.33
	Basal cell adenoma (n = 60 images from 1 case)	100.00	78.33	96.67	100.00	95.00	93.33	100.00
	Warthin’s tumor (n = 60 images from 1 case)	100.00	100.00	71.67	100.00	100.00	90.00	100.00
	Carcinoma ex pleomorphic adenoma (n = 60 images from 1 case)	75.00	66.67	68.33	96.67	98.33	80.00	71.67
	Mucoepidermoid carcinoma (n = 120 images from 2 cases)	81.24	90.17	52.71	78.63	89.09	82.98	80.08
	Adenoid cystic carcinoma (n = 120 images from 2 cases)	97.50	100.00	90.00	80.00	85.83	86.67	97.50
	Acinic cell carcinoma (n = 120 images from 2 cases)	81.67	97.50	89.17	90.83	85.83	69.17	97.50
	Salivary duct carcinoma (n = 60 images from 1 case)	100.00	100.00	58.33	100.00	100.00	76.67	100.00
	Average	84.84	90.75	73.78	87.64	88.84	81.73	90.92
Out-of-domain-C	Adenoid cystic carcinoma (n = 180 images from 3 cases)	94.44	92.22	80.00	86.11	91.11	81.11	95.56
	Basal cell adenoma (n = 180 images from 3 cases)	97.18	89.94	72.90	69.58	73.27	75.99	88.44
	Odontogenic myxoma/myxofibroma (n = 180 images from 3 cases)	95.00	100.00	76.67	92.78	98.33	98.33	93.33
	Fibrous dysplasia (n = 120 images from 2 cases)	85.83	100.00	99.17	77.50	70.00	70.00	72.50
	Osteoma (n = 180 images from 3 cases)	97.78	98.89	100.00	82.22	100.00	99.44	99.44
	Odontogenic keratocyst (n = 120 images from 2 cases)	80.00	97.50	10.83	56.67	55.83	80.83	91.67
	Orthokeratinized odontogenic cyst (n = 120 images from 2 cases)	69.17	95.00	0.83	75.83	78.33	68.33	86.67
	Adenomatoid odontogenic tumor (n = 120 images from 2 cases)	89.17	93.33	95.83	98.33	94.17	59.17	80.83
	Average	88.57	95.86	67.03	79.88	82.63	79.15	89.68

Table A5

**Table 6:** Mean-HI (SD) for each test query category for in- and out-of-domain image queries. The SSL models consistently best retrieved the histologic similarity of the results. It is noteworthy that a lower Mean-HI is preferable. The lowest Mean-HI for each category is marked in *italics*. SSL: self-supervised learning; HI: histologic inaccuracy.

Query	Category	Pre-trained VGG16	Pre-trained DINOv2	Fine-tuned ResNet18	ResNet18 + SimCLR	ResNet18 + TiCo	Ciga model	Phikon
In-domain (n = 120 images from 2 cases)	Nasopalatine duct cyst	6.93 (0.37)	6.11 (0.91)	6.03 (0.46)	4.91 (1.33)	3.90 (1.02)	6.03 (0.46)	6.13 (1.00)
	Glandular odontogenic cyst	6.00 (0.80)	6.06 (0.77)	5.81 (0.95)	4.94 (0.71)	4.79 (0.81)	5.81 (0.95)	5.11 (1.15)
	Odontogenic keratocyst	6.03 (0.98)	3.10 (0.44)	5.24 (0.53)	1.63 (0.77)	1.73 (0.95)	5.24 (0.53)	1.91 (0.54)
	Orthokeratinized odontogenic cyst	5.88 (1.38)	2.95 (0.96)	5.53 (1.40)	1.33 (1.55)	1.06 (1.18)	5.53 (1.40)	2.78 (0.82)
	Basal cell adenoma	1.53 (0.74)	3.98 (1.51)	2.51 (0.96)	1.66 (0.94)	1.24 (0.71)	2.51 (0.96)	1.51 (0.64)
	Adenoid cystic carcinoma	5.08 (1.18)	4.79 (0.72)	6.30 (1.91)	2.60 (0.77)	2.17 (0.79)	6.30 (1.91)	4.06 (2.27)
	Mucoepidermoid carcinoma	4.84 (0.88)	5.90 (1.00)	7.24 (0.98)	5.35 (1.58)	5.28 (1.84)	7.24 (0.98)	4.73 (1.16)
	Warthin’s tumor	3.01 (1.11)	3.66 (2.38)	4.38 (1.12)	1.01 (0.58)	1.58 (1.00)	4.38 (1.12)	1.62 (1.04)
	Odontogenic fibroma	6.67 (1.59)	6.66 (1.25)	7.54 (1.07)	7.03 (2.07)	7.05 (2.10)	7.54 (1.07)	7.21 (1.59)
	Ameloblastoma	7.92 (1.05)	7.83 (0.96)	8.16 (0.75)	5.98 (1.97)	6.63 (1.50)	8.16 (0.75)	7.93 (0.88)
	Hemangioma	6.32 (1.11)	7.13 (1.22)	6.40 (1.05)	4.53 (1.12)	4.78 (1.24)	6.40 (1.05)	5.33 (1.68)
	Average	5.47 (1.82)	5.29 (1.68)	5.92 (1.56)	3.72 (2.13)	3.65 (2.20)	5.92 (1.56)	4.39 (2.44)
Out-of-domain-phonecam (n = 54 images from 2 cases)	Nasopalatine duct cyst	7.83 (0.52)	5.96 (0.98)	7.30 (0.68)	4.96 (0.60)	4.24 (0.32)	6.78 (0.98)	6.67 (1.00)
	Glandular odontogenic cyst	6.48 (1.06)	5.69 (0.66)	6.52 (1.36)	4.91 (1.19)	5.04 (0.82)	5.30 (0.63)	5.44 (0.54)
	Odontogenic keratocyst	5.33 (1.11)	3.20 (1.20)	7.87 (0.61)	2.33 (1.70)	2.35 (1.58)	3.78 (0.50)	4.15 (0.79)
	Orthokeratinized odontogenic cyst	5.43 (1.50)	4.83 (1.31)	6.65 (0.79)	2.76 (2.25)	3.06 (2.41)	5.24 (1.49)	4.41 (1.14)
	Basal cell adenoma	3.28 (0.82)	5.26 (0.66)	8.65 (0.61)	2.54 (0.70)	3.67 (0.83)	4.39 (0.67)	4.89 (0.73)
	Adenoid cystic carcinoma	6.91 (1.58)	5.56 (1.46)	9.06 (0.51)	5.35 (1.96)	5.59 (2.34)	7.35 (1.81)	5.96 (1.49)
	Mucoepidermoid carcinoma	5.61 (0.83)	7.13 (1.21)	8.37 (0.68)	6.61 (1.05)	6.65 (1.15)	8.76 (0.39)	8.50 (0.72)
	Warthin’s tumor	5.74 (1.83)	4.69 (2.28)	8.65 (0.75)	2.28 (1.51)	2.26 (1.06)	6.81 (1.81)	7.69 (1.45)
	Odontogenic fibroma	8.65 (0.75)	7.63 (0.90)	9.59 (0.41)	7.98 (1.05)	7.78 (1.38)	6.78 (0.44)	8.80 (0.54)
	Ameloblastoma	8.13 (1.32)	7.00 (0.83)	7.26 (0.59)	4.96 (1.65)	6.22 (1.32)	7.80 (0.80)	6.64 (0.50)
	Hemangioma	7.33 (0.65)	7.69 (1.15)	8.24 (1.60)	6.59 (1.91)	6.46 (1.21)	7.46 (1.16)	7.33 (0.80)
	Average	6.43 (1.55)	5.88 (1.39)	8.01 (0.99)	4.66 (1.96)	4.85 (1.87)	6.40 (1.53)	6.39 (1.75)
Out-of-domain-B	Myoepithelioma (n = 60 images from 1 case)	5.88 (1.16)	5.58 (0.49)	5.98 (0.18)	5.67 (0.68)	4.55 (0.44)	6.17 (0.84)	5.28 (1.40)
	Basal cell adenoma (n = 60 images from 1 case)	3.63 (2.04)	4.57 (0.36)	3.82 (1.96)	1.70 (0.09)	1.90 (0.61)	3.73 (0.75)	1.82 (0.08)
	Warthin’s tumor (n = 60 images from 1 case)	3.55 (1.26)	2.23 (0.58)	7.70 (2.46)	1.12 (0.26)	1.75 (0.35)	7.07 (0.19)	2.72 (0.87)
	Carcinoma ex pleomorphic adenoma (n = 60 images from 1 case)	5.45 (0.64)	5.22 (1.81)	7.17 (1.07)	3.68 (0.98)	4.07 (0.58)	4.93 (1.09)	3.28 (1.64)
	Mucoepidermoid carcinoma (n = 120 images from 2 cases)	5.55 (1.41)	6.29 (0.56)	6.98 (1.24)	6.22 (1.16)	6.24 (1.34)	6.71 (0.87)	6.93 (1.11)
	Adenoid cystic carcinoma (n = 120 images from 2 cases)	2.93 (1.35)	4.03 (1.36)	5.96 (0.81)	4.38 (2.78)	4.89 (2.48)	5.88 (2.37)	4.18 (2.55)
	Acinic cell carcinoma (n = 120 images from 2 cases)	5.18 (2.50)	3.94 (2.16)	6.96 (0.88)	5.03 (1.87)	4.26 (2.21)	6.21 (2.36)	4.11 (1.35)
	Salivary duct carcinoma (n = 60 images from 1 case)	4.57 (0.53)	4.62 (0.58)	8.97 (0.68)	4.10 (0.41)	4.40 (1.02)	4.80 (2.98)	3.40 (1.78)
	Average	4.59 (1.10)	4.56 (1.23)	6.69 (1.51)	3.99 (1.80)	4.01 (1.50)	5.69 (1.11)	4.27 (2.23)
Out-of-domain-C	Adenoid cystic carcinoma (n = 180 images from 3 cases)	4.66 (1.15)	5.14 (1.29)	8.03 (0.82)	5.23 (1.69)	5.06 (1.28)	6.87 (0.67)	4.19 (0.93)
	Basal cell adenoma (n = 180 images from 3 cases)	2.90 (1.35)	5.03 (1.03)	8.81 (0.69)	5.51 (2.68)	4.65 (2.95)	5.12 (1.62)	4.62 (1.76)
	Odontogenic myxoma/myxofibroma (n = 180 images from 3 cases)	4.75 (2.36)	2.98 (1.31)	8.09 (0.82)	3.38 (1.52)	2.42 (1.54)	4.02 (1.29)	4.58 (1.67)
	Fibrous dysplasia (n = 120 images from 2 cases)	5.32 (1.02)	3.80 (0.63)	4.18 (0.35)	4.19 (0.87)	4.57 (0.70)	5.52 (0.67)	5.22 (1.14)
	Osteoma (n = 180 images from 3 cases)	3.05 (0.86)	3.32 (0.86)	3.21 (0.35)	3.08 (0.88)	3.07 (0.40)	3.59 (0.73)	2.59 (1.49)
	Odontogenic keratocyst (n = 120 images from 2 cases)	5.61 (1.80)	4.45 (0.95)	9.58 (0.68)	5.91 (2.69)	6.44 (3.06)	5.67 (2.03)	5.73 (1.92)
	Orthokeratinized odontogenic cyst (n = 120 images from 2 cases)	5.68 (1.21)	2.93 (0.46)	9.69 (0.27)	1.58 (0.85)	1.91 (1.09)	4.53 (1.45)	3.86 (2.77)
	Adenomatoid odontogenic tumor (n = 120 images from 2 cases)	5.94 (1.23)	6.76 (0.73)	6.38 (1.25)	4.78 (1.11)	5.60 (1.26)	7.48 (1.14)	5.24 (1.86)
	Average	4.74 (1.17)	4.30 (1.32)	7.25 (2.44)	4.21 (1.45)	4.21 (1.59)	5.35 (1.34)	4.40 (1.86)
